# Cell type discovery using single-cell transcriptomics: implications for ontological representation

**DOI:** 10.1093/hmg/ddy100

**Published:** 2018-03-24

**Authors:** Brian D Aevermann, Mark Novotny, Trygve Bakken, Jeremy A Miller, Alexander D Diehl, David Osumi-Sutherland, Roger S Lasken, Ed S Lein, Richard H Scheuermann

**Affiliations:** 1 J. Craig Venter Institute, La Jolla, CA 92037, USA; 2 Allen Institute for Brain Science, Seattle, WA 98109, USA; 3 Department of Biomedical Informatics, University at Buffalo, Buffalo, NY 14203, USA; 4 European Molecular Biology Laboratory, European Bioinformatics Institute, Wellcome Trust Genome Campus, Hinxton, CB10 1SD, UK; 5 Department of Pathology, University of California San Diego, La Jolla, CA 92093, USA

## Abstract

Cells are fundamental function units of multicellular organisms, with different cell types playing distinct physiological roles in the body. The recent advent of single-cell transcriptional profiling using RNA sequencing is producing ‘big data’, enabling the identification of novel human cell types at an unprecedented rate. In this review, we summarize recent work characterizing cell types in the human central nervous and immune systems using single-cell and single-nuclei RNA sequencing, and discuss the implications that these discoveries are having on the representation of cell types in the reference Cell Ontology (CL). We propose a method, based on random forest machine learning, for identifying sets of necessary and sufficient marker genes, which can be used to assemble consistent and reproducible cell type definitions for incorporation into the CL. The representation of defined cell type classes and their relationships in the CL using this strategy will make the cell type classes being identified by high-throughput/high-content technologies findable, accessible, interoperable and reusable (FAIR), allowing the CL to serve as a reference knowledgebase of information about the role that distinct cellular phenotypes play in human health and disease.

## Introduction

Cells are probably the most important fundamental functional units of multicellular organisms, since different cell types play different physiological roles in the body. Although every cell of an individual organism contains essentially the same genome structure, different cells realize diverse functions due to differences in their *expressed* genome. In many cases, abnormalities in gene expression form the physical basis of disease dispositions. Thus, understanding and representing normal and abnormal cellular phenotypes can lead to the development of biomarkers for diagnosing disease and the identification of critical targets for therapeutic interventions.

Previous approaches used to characterize cell phenotypes have several drawbacks that limited their ability to comprehensively identify the cellular complexity of human tissues. Transcriptional profiling of bulk cell sample mixtures by microarray or RNA sequencing can simultaneously assess gene expression levels and proportions of abundant known cell types, but precludes identification of novel cell types and obscures the contributions of rare cell subsets to the gene expression patterns present in the bulk samples. Flow cytometry provides phenotype information at the single cell level, but is limited by the number of discrete markers that can be assessed, and relies on prior knowledge of marker expression patterns. The recent establishment of methods for single-cell transcriptional profiling (1,2) is revolutionizing our ability to understand complex cell mixtures, avoiding the averaging phenomenon inherent in the analysis of bulk cell mixtures and providing for an unbiased assessment of phenotypic markers within the expressed genome.

In order to compare experimental results and other information about cell types, a standard reference nomenclature that includes consistent cell type names and definitions is required. The Cell Ontology (CL) is a biomedical ontology developed to provide this standard reference nomenclature for *in vivo* cell types in humans and major model organisms (3). However, the advent of high-content single-cell transcriptomics for cell type characterization has resulted in a number of challenges for their representation in the CL (discussed in 4). In this paper, we review some of the recent discoveries that have resulted from the application of single-cell transcriptomics to human samples, and propose a strategy for defining cell types within the CL based on the identification of necessary and sufficient marker genes, to support interoperable and reproducible research.

## Application to the human brain

Initial progress in neuronal cell type discovery by single-cell RNA sequencing (scRNAseq) focused on mouse cerebral, visual and somatosensory cortices (5–9). More recently, technological advances, including RNAseq using single nuclei (snRNAseq) instead of single cells (10–12), have extended these investigations into human neuronal cell type discovery (13,14). Direct comparisons of matched transcriptomic profiles generated by single-cell and single-nucleus RNAseq in mouse cortex found high concordance in cell types discovered by each method individually **(**15,16); however, some transcripts were found to be enriched in either the cytoplasm or the nucleus. Depending on the identity of the enriched transcripts, these differences may have an impact when mapping to a reference database of cells. Comprehensive reviews of these recent advances have been reported recently (17–19).

Initial efforts toward human neuronal cell type discovery focused on identifying broad lineages. Pollen *et al.* profiled 65 neuronal cells into six categories: neural progenitor cells, radial glia, newborn neurons, inhibitory interneurons and maturing neurons (20), while Darmanis *et al.* sequenced 466 cells, also identifying six broad, but distinct, categories: oligodendrocytes, astrocytes, microglia, endothelial cells, oligodendrocyte precursor cells (OPCs) and neurons (21). Darmanis *et al.* further subtyped the adult neurons into two excitatory and five inhibitory types. More recent single *nuclei* RNAseq investigations are attempting more comprehensive cell typing. Lake *et al.* sampled 3227 nuclei from six Brodmann areas, from which the neurons were classified into eight excitatory and eight inhibitory subtypes (13). Similarly, Boldog *et al.* sampled 769 nuclei from layer 1 of the middle temporal gyrus (MTG) and identified 11 distinct inhibitory cell types (14).

Comparing results between these studies has been challenging given the different areas and layers of cortex sampled. Many of the studies leveraged classical cell type markers derived from the mouse scRNAseq literature. For example, SNAP25 expression was used to broadly define neuronal cells, while GAD1 expression defined inhibitory interneurons. Additional classical markers have then been used to subdivide the excitatory and inhibitory classes, such as CUX2 or VIP respectively; however, these markers individually are still not specific enough to define discrete cell type classes at the level of granularity revealed by clustering of the sc/snRNAseq data. In fact, there has been surprisingly limited overlap in gene sets specific for individual cell type clusters between studies, as the genes found in each study appear to be sensitive to both the context and methodology used. For example, Lake *et al.* found that cluster In1 had CNR1 (Supplementary Material, Table S5 in reference 13) as the highest ranked marker, while Boldog *et al.* found seven distinct inhibitory types that expressed this marker (Fig. 3 in reference 14). Without a standardized methodology for determining the necessary and sufficient marker genes and a corresponding marker gene reference database, comparison of newly identified cell types to those reported in previous studies requires a complete reprocessing of the data.

## Application to the human immune system

Single-cell transcriptomic analysis has also been applied to study the functional cell type diversity of the human immune system (reviewed in 22). Bjorklund *et al.* used scRNAseq to explore the subtype diversity of CD127+ innate lymphoid cells isolated from human tonsil, providing an in-depth transcriptional characterization of the three major subtypes: ILC1, ILC2 and ILC3, and three additional subtypes within the ILC3 class, by comparing their single-cell transcriptional profiles (23).

Two recent studies explored the subtype diversity of dendritic cells in human blood. In addition to identifying two conventional dendritic cell subtypes (cDC1 and cDC2) and one plasmacytoid dendritic cell subtype, See *et al.* identified several subtypes that appear to correspond to precursor cells, including one early uncommitted CD123^+^ pre-DC subset and two CD45RA^+^CD123^lo^ lineage-committed subsets (pre-cDC1 and pre-cDC2), using cell sorting, scRNAseq and *in vitro* differentiation assays (24). Villani *et al.* used fluorescence-activated cell sorting and scRNAseq to delineate six different dendritic cell subtypes (DC1–6) and four different monocyte subtypes (Mono1–4), and went on to show that these different subtypes, which were defined based on their transcriptional profiles, exhibited different functional capabilities for allogeneic T cell stimulation and for cytokine production following TLR agonist stimulation (25).

Two recent studies have explored the phenotypes of immune cells infiltrating tumor specimens using scRNAseq. In melanoma, Tirosh *et al.* found that the non-malignant tumor microenvironment was composed of T cell, B cell, NK cell, endothelial cell, macrophage and cancer-associated fibroblast (CAF) subsets (26). In contrast to the distinct transcriptional phenotypes of the malignant component across individual melanoma specimens, common features could be observed in the non-malignant components, with important therapeutic implications. Expression of multiple complement factors by CAFs correlated with the extent of T cell infiltration. T cells with activation-independent exhaustion profiles, characterized by expression of co-inhibitory receptors (e.g. PD1 and TIM3), could be distinguished from cytotoxic T cell profiles. Potential biomarkers that distinguish between exhausted and cytotoxic T cells could aid in selecting patients for immune checkpoint blockade. In hepatocellular carcinoma, Zheng *et al.* found clonal enrichment of both regulatory T cells and exhausted CD8 T cells using scRNAseq and T cell receptor repertoire analysis (27). The diagnostic and prognostic significance of these findings remain to be explored.

While these studies illustrate the power of single cell genomics to identify important functional cell subtypes, they also illuminate a major challenge in comparing the results from different studies, due to the lack of a consistent, reusable approach for naming, defining and comparing new cell types being identified by these high content phenotyping technologies. For example, in the two studies focused on the identification of dendritic cell subtypes, it is unclear if the cDC1 and cDC2 subtypes identified by See *et al.* correspond to the DC1 and DC2 subtypes identified by Villani *et al.* Indeed, the only way to make this determination would be to perform a *de novo* comparative analysis of the transcriptional profiles from both studies. For these studies to truly comply with the newly emerging FAIR principles of open data (28), a robust reproducible strategy for defining and representing new cell types is essential to support their broad interoperability.

## Application to other tissue types

Recent advances in cell type discovery by single-cell or single-nuclei RNAseq have not been isolated to the fields of neurology or immunology. Preliminary investigations have also been made to characterize the cell types in kidney (29), lung (30) and pancreas (31–34) (Table 1), with more on the way.

**Table 1.** Model tissues investigated by single-cell/single-nuclei RNA sequencing

Tissue	Number of cell types	Method	Reference
Brain	6 cell categories	Single-cell RNAseq	(20)
Brain	7 neuron subtypes	Single-cell RNAseq	(20)
Brain	16 neuron subtypes	Single-nuclei RNAseq	(13)
Brain	11 inhibitory neuron subtypes	Single-nuclei RNAseq	(20)
Immune system	5 CD127+ subtypes	Single-cell RNAseq	(23)
Immune system	6 dendritic cell subtypes	Single-cell RNAseq	(24)
Immune system	6 dendritic cell and 4 monocyte subtypes	Single-cell RNAseq	(25)
Tumor microenvironment	6 infiltrating immune subsets	Single-cell RNAseq	(26)
Tumor microenvironment	Regulartory T cells and exhausted CD8 T cells	Single-cell RNAseq	(27)
Kidney	6 distinct epithelial subtypes	Single-nuclei RNAseq	(29)
Lung	4 cell types (C1–C4): AT2, indeterminate, basal and club/goblet cells	Single-cell RNAseq	(30)
Pancreas	6 cell types (alpha, beta, delta, PP, acinar or ductal)	Single-cell RNAseq	(31)
Pancreas	6 cell types (alpha, beta, delta, PP, acinar or ductal)	Single-cell RNAseq	(32)
Pancreas	14 cell types including known exocrine and endocrine types	Single-cell RNAseq	(33)
Pancreas	9 cell types including known exocrine and endocrine types	Single-cell RNAseq	(34)

## Ontological representation

Biomedical ontologies, as promoted by the Open Biomedical Ontology (OBO) Foundry (35), provide for a framework to name and define the types, properties and relationships of entities in the biomedical domain. The CL was established in 2005 to provide a standard reference nomenclature for *in vivo* cell types, including those observed in specific developmental stages in humans and different model organisms (3). The semantic hierarchy of CL is mainly constructed using two core relations: *is_a* and *develops_from*. Masci *et al.* proposed a major revision to the CL using dendritic cells as the driving biological use case in which the expression of specific marker proteins on the cell surface (e.g. receptor proteins) or internally (e.g. transcription factors) would be used as the main *differentia* for the asserted hierarchy (36). Diehl *et al.* applied this approach first to cell types of the hematopoietic system and then later to the full CL (37–39). As of December 2017, the CL contained 2199 cell type classes, with 583 classes within the hematopoietic cell branch alone.

We recently discussed some of the challenges faced by the CL in the era of high-throughput, high-content single-cell phenotyping technologies, including sc/snRNAseq (4). One of the key recommendations was to establish a standard strategy for defining cell type classes that combine three essential components:

- The minimum set of ***necessary and sufficient marker genes***selectively expressed by the cell type
- A ***parent cell class***in the CL
- A ***specimen source description***(anatomic structure + species).

In order to identify the set of necessary and sufficient marker genes from an sc/snRNAseq experiment, we have developed a method—NSforest—that utilizes a random forest of decision trees machine learning approach. The methodology described here is unique in that it determines the *minimum number of differentiallyexpressed genes*, working in concert, that are sufficient to define a cell type from a given dataset. These marker genes can then be used for a variety of purposes, including the construction of semantic definitions in an ontological context. Table 2 lists other methods that can be used for the identification of all cell cluster-specific differentially expressed genes (6,40,41).

**Table 2.** Additional tools for deterimination of cell type-specific differentially expressed genes

Software	Methodology	Reference
Seurat	Seurat implements numerous methodolgies for clustering, visualization and marker determination using differential expression analysis between cluster pairs	(6)
SC3	SC3 provides an integrated suite that performs an ensemble clustering followed by marker determination using a Wilcoxon signed ranked test combined with an AUROC analysis	(40)
SAKE	SAKE performs a negative matrix factorization (NMF) where the importance of a given cell and gene are estimated during the clustering procedure, these important genes are then considered markers	(41)

**Figure 1.**
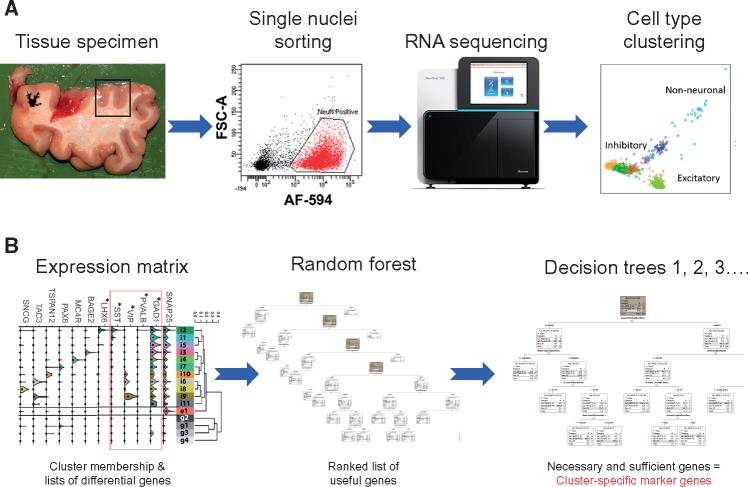
Identification of necessary and sufficient marker genes using NSforest. (**A**) A typical single-cell/single-nuclei RNA sequencing workflow in which a tissue specimen is obtained, single cells/nuclei isolated by fluorescence-activated cell sorting, amplified cDNA processed by sequencing and cell types identified by clustering the resultant transcriptional profiles. (**B**) The NSforest approach takes a data matrix of expression values (e.g. transcripts per million reads) of genes (rows) in single cell/nuclei samples (columns) grouped by cell type cluster membership. In the first step, the expression levels of genes are used as features in the random forest machine learning procedure to train classification models comparing single cell/nuclei expression data in one cell type cluster against single cell/nuclei expression data in all other clusters, for every cell type cluster separately, using a Random Forest Learner like KNIME v3.1.2. Each cell type cluster classification model is constructed from a collection of trees (e.g. 1000 trees) using information gain ratio as the splitting criteria, where each decision tree is generated using the specific bagging parameters (e.g. the square root of the number of features and a bootstrap of samples equal to the training set size). For each cell type cluster classification model, the method outputs usage statistics, including how often each gene is used as a branching criterion and the number of times it was a candidate across all random decision trees. By summing the frequency of use when available as a candidate feature along the first three branching levels, the list of genes can be ranked by their usefulness in distinguishing one cell type cluster from the other clusters. In the second step, single decision trees are constructed using the first gene from the ranked list, the first two genes, the first three genes, etc. Each individual tree is then assessed for classification accuracy and tree topology using the training data. Given the objective of determining the necessary and sufficient marker genes, we apply additional criteria in scoring the trees—we restrict each gene to being used in only one branch per tree, and find the optimal classification for the target cluster only, rather than the overall classification score. The addition of genes from the ranked list is stopped when an optimal classification or stable tree topology is achieved. The minimum number of genes used to produce this optimal result corresponds to the set of necessary and sufficient marker genes required to define the cell type cluster.

To illustrate how this approach can produce standard cell type definitions, we have applied the method to a transcriptomic dataset derived from single nuclei isolated from the MTG, cortical layer 1 of a post-mortem human brain specimen (Fig. 1A in reference 14). Transcriptional profiles obtained from RNA sequencing of a collection of single sorted nuclei was used to identify 16 discrete cell types using an iterative data clustering approach. Based on the expression of the previously characterized marker genes SNAP25 and GAD1 for broad classes, 11 inhibitory interneurons, 1 excitatory neuron and 4 glial cell type clusters were identified.

In the first step (Fig. 1B), NSforest takes the gene expression data matrix of genes versus single nuclei with their cell type cluster membership as input. The gene expression data matrix and cluster memberships are supplied by the user. Consequently, issues related to requirements for data normalization to control for batch effects, data filtering to remove poor quality samples, controlling for cell cycle effects and the effects of the clustering methodology selected need to be carefully considered to ensure robust cluster membership and thereby informative marker genes. With these inputs, a classification model is developed for each cell type cluster by comparing each Cluster X versus all non-Cluster X profiles using the Random Forest algorithm (42). In addition to the classification model itself, NSforest produces a ranked list of features (genes) that are most informative for distinguishing between Cluster X and all of the other clusters.

In the second step, NSforest constructs single decision trees using first the top gene, then the top two genes, top three genes, etc., until a stable tree topology and optimal classification accuracy is achieved. The minimum number of genes necessary to obtain this stable classification result corresponds to the necessary and sufficient set of marker genes defining each cell type cluster within this experimental context.

**Figure 2.**
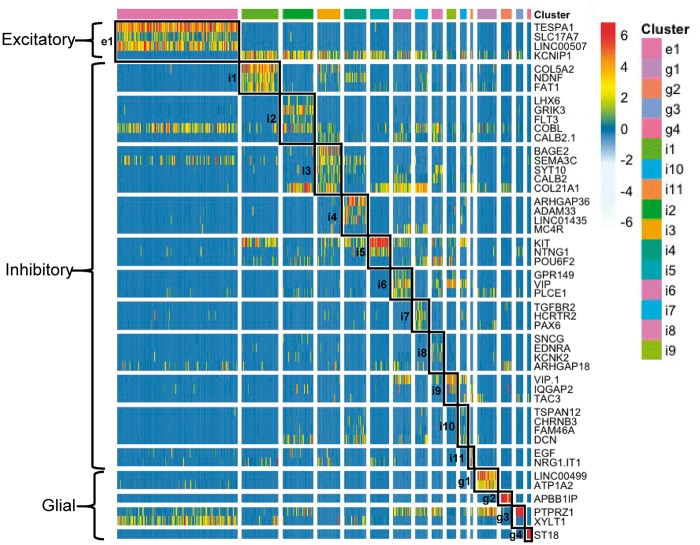
Marker gene expression patterns in single nuclei grouped by cluster. A heatmap of expression levels for the necessary and sufficient marker genes identified for all 16 clusters across all single nuclei grouped by cell type cluster is shown, including 1 excitatory (e1), 11 inhibitory (i1–i11) and 4 glial (g1–g4) cell type clusters. In total, 49 markers genes were selected as being necessary and sufficient to distinguish these 16 different cell type clusters from cortical layer 1/2 of the human brain MTG region.

The expression of the complete set of marker genes obtained from applying NSforest to the single nuclei dataset is illustrated in Figure 2. In most cases, the expression of three marker genes is sufficient to define a cell type cluster, with a range of one to five necessary and sufficient marker genes per cluster. Glial cell subtypes appear to be more distinct from each other, requiring relatively few genes to sufficiently define the cell type. In contrast, neuronal subtypes appear to be more similar, requiring more genes to achieve specificity. In some cases, a combination of both positive and negative expression optimally defines a cell type cluster.

For one of the inhibitory interneuron cell types defined in this study (i5), we were able to connect the distinct transcriptional profile with a previous cell type defined based on its unique cellular morphology—the Rosehip cell (14). This then allows us to construct an ontological representation that includes both a colloquial name, an alternative name and a definition combining the necessary and sufficient marker genes, a CL parent cell class and specimen source information, as follows:

- Colloquial name—***rosehip neuron***
- Alternative name—***KIT-expressing MTG cortical layer 1 GABAergic interneuron, human***
- Definition—***A humanMTGcortical layer 1 GABAergic interneuron that selectively expresses KIT, NTNG1and POU6F2 mRNAs***

A complete set of cell type names and definitions for all cell type clusters identified in this experiment is provided in Table 3.

**Table 3.** Cell types identified in cortical layer 1/2 of the human MTG

Cluster ID	Cell type name	Cell type definition
**e1**	TESPA1-expressing MTG cortical layer 2 excitatory neuron, human	A human MTG cortical layer 2 excitatory neuron that selectively expresses TESPA1, LINC00507 and SLC17A7 mRNAs, and lacks expression of KCNIP1 mRNA
**i1**	COL5A2-expressing MTG cortical layer 1 interneuron, human	A human MTG cortical layer 1 GABAergic interneuron that selectively expresses COL5A2 and NDNF and FAT1 mRNAs
**i2**	LHX6-expressing MTG cortical layer 2 interneuron, human	A human MTG cortical layer 2 GABAergic interneuron that selectively expresses LHX6, GRIK3 and FLT3, while of lacking expression of COBL and CALB2 mRNAs
**i3**	BAGE2 expressing MTG cortical layer 1 interneuron, human	A human MTG cortical layer 1 GABAergic interneuron that selectively expresses BAGE2 and SEMA3C and SYT10 and CALB2 and COL21A1 mRNAs
**i4**	ARHGAP36 expressing MTG cortical layer 1 interneuron, human	A human MTG cortical layer 1 GABAergic interneuron that selectively expresses ARHGAP36 and ADAM33 and LINC01435 and MC4R mRNAs
**i5**	KIT-expressing MTG cortical layer 1 interneuron, human	A human MTG cortical layer 1 GABAergic interneuron that selectively expresses KIT and NTNG1 and POU6F2 mRNAs
**i6**	GPR149-expressing MTG cortical layer 1 interneuron, human	A human MTG cortical layer 1 GABAergic interneuron that selectively expresses GPR149 and VIP and PLCE1 mRNAs
**i7**	TGFBR2-expressing MTG cortical layer 1 interneuron, human	A human MTG cortical layer 1 GABAergic interneuron that selectively expresses TGFBR2 and HCRTR2 and PAX6 mRNAs
**i8**	SNCG-expressing MTG cortical layer 1 interneuron, human	A human MTG cortical layer 1 GABAergic interneuron that selectively expresses SNCG and EDNRA and KCNK2 and ARHGAP18 mRNAs
**i9**	VIP-expressing MTG cortical layer 1 interneuron, human	A human MTG cortical layer 1 GABAergic interneuron that selectively expresses VIP and IQGAP2 and TAC3 mRNAs
**i10**	TSPAN12-expressing MTG cortical layer 1 interneuron, human	A human MTG cortical layer 1 GABAergic interneuron that selectively expresses TSPAN12 and CHRNB3 and FAM46A and DCN mRNAs
**i11**	EGF-expressing MTG cortical layer 1 interneuron, human	A human MTG cortical layer 1 GABAergic interneuron that selectively expresses EGF and NRG1-IT1 mRNAs
**g1**	Linc00499-expressing MTG cortical layer 1 glial cell, human	A human MTG cortical layer 1 glial cell that selectively expresses Linc00499 and ATP1A2 mRNAs
**g2**	APBB1IP-expressing MTG cortical layer 1 glial cell, human	A human MTG cortical layer 1 glial cell that selectively expresses APBB1IP mRNAs
**g3**	PTPRZ1-expressing MTG cortical layer 1 glial cell, human	A human MTG cortical layer 1 glial cell that selectively expresses PTPRZ1 and XYLT1 mRNAs
**g4**	ST18-expressing MTG cortical layer 1 glial cell, human	A human MTG cortical layer 1 glial cell that selectively expresses ST18 mRNAs

**Figure 3.**
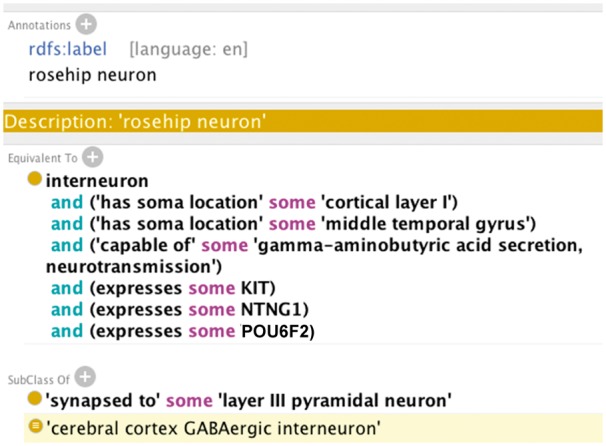
Formal rosehip neuron definition using logical axioms. A set of logical axioms about the anatomical location of the cell body (soma), the functional capacity and the necessary and sufficient marker gene expressions are combined to construct an equivalent class cell type definition for the rosehip neuron interneuron cluster—i5 (see 14 for more information about how this cell type was characterized).

These informal textual definitions can then be converted into formal ontological definitions, represented in OWL as equivalent classes, using a set of logical axioms that combine assertions about the parent cell class (interneuron), anatomic locations of the neuron cell body (soma), functional capacity of the cell type (gamma-aminobutyric acid secretion) and marker gene expression (expresses some KIT) requirements (Fig. 3). Using semantic reasoners, these logical axioms can then be used to infer novel characteristics, e.g. SubClass Of ‘cerebral cortex GABAergic interneuron’.

The challenge remains of ensuring that these cell type definitions, whose necessary and sufficient conditions are derived from analysis of data from one particular methodology (scRNAseq), are compatible with both existing cell type classes in the CL and cell types defined using alternative experimental methods and data analysis approaches. Working with CL developers, we are now establishing an extension ontology module containing provisional definitions for novel cell types that we and other research groups will contribute. Ontological reasoners will be used to link these cell types to more general classes in the CL proper, structure them into an extended hierarchy, and determine when separate research groups have defined similar or identical cell types. CL developers will review these provisional cell types periodically to determine when multiple lines of evidence provide sufficient support to promote particular cell type classes to the CL itself. In this way we will ensure the integrity of the CL reference, while still allowing for the rapid expansion of its content to accommodate cell types defined via these new technologies. However, it should be noted that defining cell types will likely be an iterative process where *in situ* validation and multi-modal data acquisition will guide refinement of cell type definitions. This review shows a path for defining cell type markers that can be used for these validations and will help guide these refinements.

## Conclusions

The application of high-throughput/high-content cytometry and single-cell genomic techniques is producing an explosion in the number of distinct cellular phenotypes being identified in human specimens. For biomedical ontologies to stay relevant, it will be critical for ontology developers to establish procedures for the processing and incorporation of representations derived from these data-intensive technologies into reference ontologies in a timely fashion. The representation of defined cell types and their relationships in the CL will serve as a reference knowledgebase to support interoperability of information about the role of cellular phenotypes in human health and disease.

*Conflict of Interest statement* **.** None declared.

## Funding

This work was supported by the Allen Institute for Brain Science, the JCVI Innovation Fund, the U.S. National Institutes of Health (R21-AI122100 and U19-AI118626), the California Institute for Regenerative Medicine (GC1R-06673-B), the Wellcome Trust 208379/Z/17/Z and from the Chan Zuckerberg Initiative DAF, an advised fund of the Silicon Valley Community Foundation (2018–182730). We thank Nik Schork, Jamison McCorrison, Pratap Venepally, Lindsay Cowell, Bjoern Peters, and Sirarat Sarntivijai for helpful discussion. Funding to pay the Open Access publication charges for this article was provided by the Chan Zuckerberg Initiative DAF, an advised fund of the Silicon Valley Community Foundation (2018-182730).
